# Monitoring stress and allostatic load in first responders and tactical operators using heart rate variability: a systematic review

**DOI:** 10.1186/s12889-021-11595-x

**Published:** 2021-09-18

**Authors:** Sean L. Corrigan, Spencer Roberts, Stuart Warmington, Jace Drain, Luana C. Main

**Affiliations:** 1 Deakin University, Centre for Sport Research, School of Exercise and Nutrition Sciences, 221 Burwood Highway, Burwood, Victoria 3125 Australia; 2 Deakin University, Institute for Physical Activity and Nutrition, Geelong, Victoria Australia; 3 Defence Science and Technology Group, Fishermans Bend, Australia

**Keywords:** Autonomic regulation, Police, Recovery, Soldiers, Decision-making

## Abstract

**Background:**

Awareness of the cumulative stress placed on first responders and tactical operators is required to manage acute fatigue, which can impair occupational performance, and may precipitate negative chronic health outcomes. The aim of this review was to investigate the utility of heart rate variability (HRV) to monitor stress and allostatic load among these populations.

**Methods:**

A systematic search of Academic Search Complete, MEDLINE complete, PsycINFO, SPORTDiscus and Scopus databases was conducted. Eligibility criteria: original peer reviewed research articles, written in English, published between 1985 and 2020, using human participants employed as a first responder or tactical operator, free from any psychological disorder.

**Results:**

Of the 360 articles screened, 60 met the inclusion criteria and were included for full text assessment. Articles were classified based on single or repeated stressor exposure and the time of HRV assessment (baseline, during stressor, post stressor). Singular stressful events elicited a reduction in HRV from baseline to during the event. Stressors of greater magnitude reduced HRV for extended durations post stressor. Lower resting HRV was associated with lower situational awareness and impaired decision-making performance in marksmanship and navigation tasks. There were insufficient studies to evaluate the utility of HRV to assess allostatic load in repeated stressor contexts.

**Conclusion:**

A reduction in HRV occurred in response to acute physical and cognitive occupational stressors. A slower rate of recovery of HRV after the completion of acute occupational stressors appears to occur in response to stressors of greater magnitude. The association between lower HRV and lower decision-making performance poses as a useful tool but further investigations on within subject changes between these factors and their relationship is required. More research is required to investigate the suitability of HRV as a measure of allostatic load in repeated stress exposures for fatigue management in first responder and tactical operators.

**Supplementary Information:**

The online version contains supplementary material available at 10.1186/s12889-021-11595-x.

## Background

Workers in physically and cognitively demanding occupations include emergency first responders (firefighters, paramedics), and tactical operators (law enforcement, military). These personnel are often required to complete physiologically demanding tasks that may be performed repeatedly over a long duration [1, 2]. Furthermore, first responders and tactical operators are exposed to the emotional toll of life and death decisions that may exacerbate the effects of physiological, cognitive and psychological stressors they experience [3, 4]. While stress forms the cornerstone of adaption, repeated exposure to stress without sufficient recovery results in cumulative fatigue and exhaustion [3]. As a result, fatigue can impair performance in occupationally relevant tasks including decision making capability [4, 5]. For example, soldiers exhibiting fatigue have demonstrated increased error of omission rates (failure to shoot when appropriate) in shooting based scenarios [4]. In addition, the accumulated stress exposure across an individual’s career may precipitate negative health outcomes if not adequately managed [6]. In particular, the prevalence of post-traumatic stress disorder [7] and some cancers [8] is higher in tactical operators and emergency service personnel when compared with general population. Therefore, methods for assessing the effects of allostatic load, the cost of chronic exposure to physical and psychological stressors, is required to better optimise health and performance in these populations [9].

Managing the stress exposure and fatigue of tactical operators and first responders proves difficult due to the multifaceted nature of these occupations. There is a growing requirement for markers that can assist in identifying when individuals are ready for duty that not only ensures their health and wellbeing is maintained, but also their occupational performance. Hormonal and self-report measure have previously been used to assess allostatic load in tactical operators and first responders [3, 10]. Some blunted responses of growth hormone, prolactin, cortisol and adrenocorticotropic hormone have presented in response to acute physical exercise in physically overloaded individuals [10, 11]. However, basal hormonal measures do not appear to provide a clear indication of allostatic load [11]. Furthermore, monitoring blood and saliva biomarkers is costly and time intensive, particularly when performing exercise tests [12], making it unsuitable for ongoing monitoring in these large scale populations. Self-report measures, which are used widely in high performance sport [13], have demonstrated relationships between mood disturbances, acute stressors and sustained allostatic load [10, 14]. In particular, subjective ratings of depressed mood and recovery as well as increases ratings of fatigue and stress have been observed in periods of increased allostatic load (i.e. increased physical training and sleep restriction) [10, 14]. It’s suggested that combining subjective and objective measures together provides a complementary measure for individual monitoring that can help discern individual responses to stress [10, 15]. Therefore, candidate objective measures of allostatic load that can accompany self-report measures in readiness monitoring in occupational settings requires further investigation.

Heart rate variability (HRV), the variation in inter-beat intervals of the heart represents the level of activation of the autonomic nervous system and may reflect the level of physiological and psychological stress an individual is experiencing [16, 17]. HRV can be used to assess the balance between sympathetic and parasympathetic contributions to the heart rhythm that result from both feedforward (i.e. motor cortex) and feedback (i.e. baroreflex) inputs to the cardiovascular control centre [18]. The use of HRV in first responders and tactical operators has shown to be sensitive to particular work characteristics of the occupations. For example, the gravitational effects of being upright promote sympathetic dominance over recumbent positions [19, 20], greater parasympathetic and reduced sympathetic activity is observed during the night (6 pm-6 am) [21] and nocturnal shift work exhibits reduced sympathetic activation when compared to morning and evening shifts [22]. If HRV is sensitive to the magnitude of physiological and psychological stressors that first responders and tactical operators experience, it poses as a potentially useful tool in monitoring [23, 24]. However, to calculate HRV there are a range of different assessment protocols (e.g. supine, standing, and 24 h recordings) and analysis techniques (time domain analysis, frequency domain analysis, and non-linear analysis), which can make interpreting HRV difficult when comparing studies that utilise different methods [17]. The aim of the current review was to better understand how HRV parameters have been used in occupational contexts for monitoring an individual’s allostatic load, their recovery, and to identify whether HRV is a suitable tool for the monitoring of stress and fatigue in these occupational contexts. In doing so, we may better understand the balance between stress exposure, recovery, and when first responders and tactical operators are ready to return to duty.

## Methods

This systematic review conformed to the Preferred Reporting Items for Systematic Reviews and Meta-Analyses (PRISMA) [25].

### Search strategy

An electronic search was conducted using the electronic databases Academic search complete, Medline complete, PsycINFO, PubMed, Scopus and SPORTDiscus. Titles and abstracts were searched for combinations of the following terms with an ‘*’ indicating a truncation: ambulance personnel, armed forces, army, defence force*, emt, firefighters, fire fighters, fire-fighters, first respond*, air force, navy, paramedic*, police*, law enforcement, military, soldiers, troops, rescue worker*, rescue-worker*, coast guard, coast-guard, emergency, and heart rate variability, hrv, ans, autonomic, pns, parasympathetic, sns, sympathetic, vagal, and fatigue*, load*, stress, overtrain*, overreach*, recovery (full searches detailed in additional file 1). Databases were searched from 1985 until 3rd August 2020 and were required to meet the following inclusion criteria: 1) the study recruited human participants that were currently employed as a first responder, tactical operator or completing the task(s) of these occupations (detailed in search terms); 2) the study reported on heart rate variability at ≥1 timepoint; 3) the study included a stressor that was applied to participants; 4) participants included in the study had no presence of psychological disorders; 5) the study was peer-reviewed and written in the English language. Studies that included measures of HRV in populations with psychological disorders (e.g. post-traumatic stress disorder) were removed due to the complexity of the disorders and the desire to focus on the utility of HRV to monitor stress, adaptation and fatigue in first responders and emergency service personnel. Additionally, reference lists of included studies were searched for relevant studies that met the inclusion criteria.

### Study selection

**Fig. 1:**
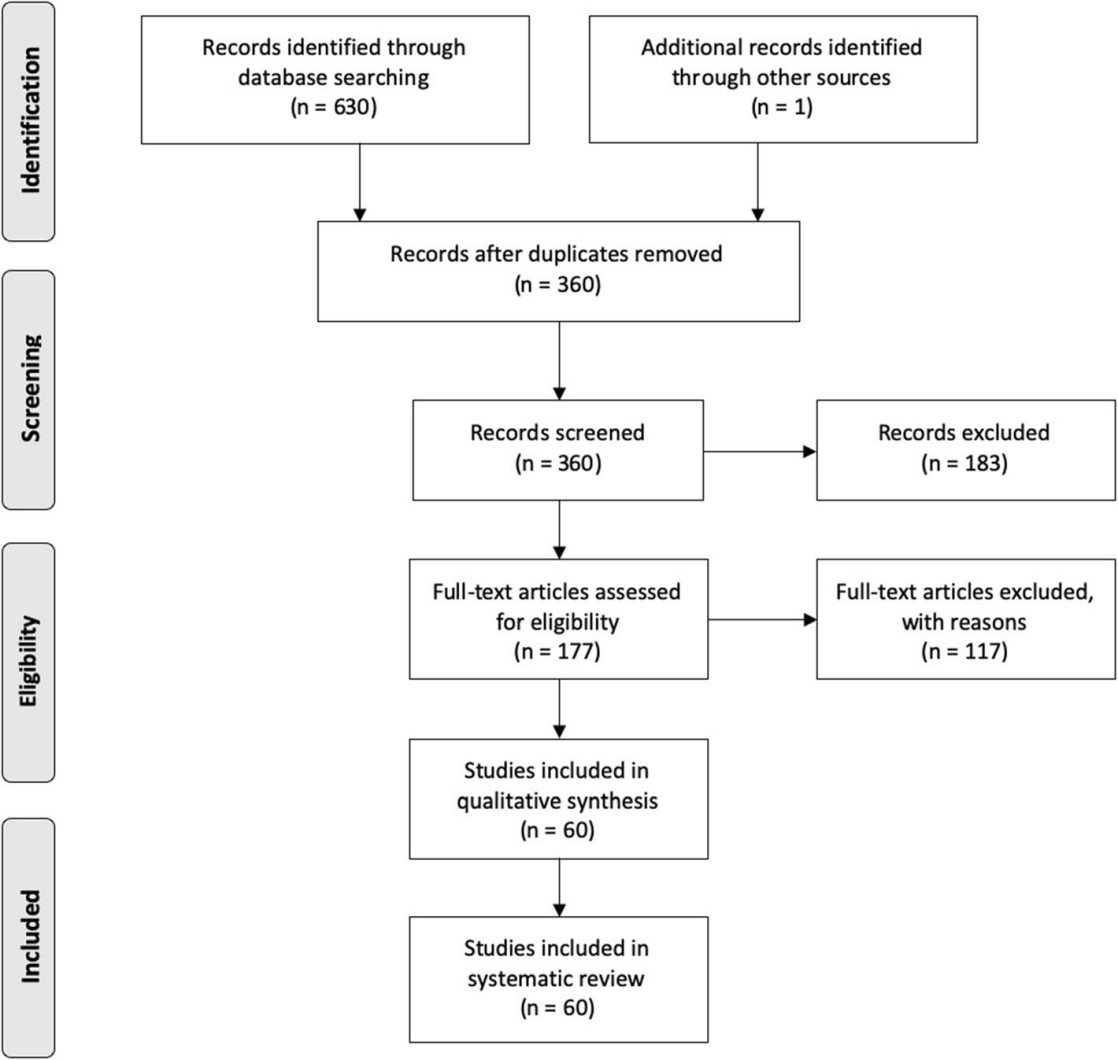
PRISMA selection of studies flow chart

Articles were first screened by title and abstract and proceeded to full text assessment if no contradiction to the inclusion criteria was present (see Fig. 1). Articles were independently assessed against the inclusion criteria by the first (SC) and last author (LM) at both stages. Discrepancies between inclusion assessments were resolved by the second author’s (SR) evaluation.

### HRV metrics

Of measures the are used to assess HRV, the following were utilised for the review: R-R interval (RRi), root mean square of successive differences of normal R-R intervals (RMSSD), standard deviation of normal R-R intervals (SDNN), percentage of normal R-R intervals greater than 50 ms (PNN50), total power (TP), very low frequency power (VLF), low frequency power (LF), high frequency power (HF), low frequency normative units (LF n.u.), high frequency normative units (HF n.u.), low frequency to high frequency ratio (LF/HF ratio), standard deviation of the distance of data from the line of identity on a poincairé plot (SD1), standard deviation of the distance of data perpendicular to the line of identity at the average RRi length on a poincairé plot (SD2) [17, 26]. The physiological mechanisms underpinning VLF (0.003–0.04 Hz) is still not fully understood but has shown associations with negative health outcomes [17]. HF (0.15–0.4 Hz) is deemed an indicator of parasympathetic modulation while LF (0.04–0.15 Hz) is thought to reflect a combination of sympathetic and parasympathetic modulation [18]. As such TP (< 0.4 Hz) reflects the combined modulation of sympathetic and parasympathetic branches [18]. LF n.u. and HF n.u. are the % proportions of LF and HF power respectively within their combined frequency band (0.04–0.4 Hz). As such, LF n.u. are thought to reflect sympathetic modulation as any changes in parasympathetic modulation will affect both LF and HF simultaneously [17, 18]. Furthermore, the LF/HF ratio, LF n.u. and HF n.u. have been used to indicate the balance between sympathetic and parasympathetic modulation [17, 18]. Of the time and non-linear metrics, RMSSD, PNN50 and SD1 are seen to reflect short term HRV while SDNN and SD2 reflect long term HRV [17, 18, 26].

### Evidence quality appraisal

As no suitable published assessment criteria were available, the methodological quality of each included study was assessed using an adapted version of the Newcastle-Ottawa Scale [27] (detailed in additional file 2). The methodological quality assessment considered the number of participants and participant details, their occupation status, how HRV was measured, and clarity around the reporting of the stressor exposure. Each subscale item was awarded a score according to the extent to which the item had been satisfied (i.e. 0 = not satisfied, 1 = partially satisfied, 2 = adequately satisfied). Discrepancies were discussed between the first (SC) and second author (SR) until a consensus was reached. The sum of the subscale item scores was used to provide an overall assessment of evidence quality up to a maximum possible score of 14 (i.e., > 11 = high quality, 8–11 = moderate quality, < 8 = low quality, detailed in additional file 3). No risk of bias was undertaken at the outcome level because all studies included an objective measure of HRV.

## Results

### Search results

A total of 631 records were retrieved from the initial search. After duplicates were removed, 177 full text articles were assessed for eligibility, from which 60 met the inclusion criteria and were included in the systematic review (see Fig. 1). Of the 60 included studies examined, participant populations were from defence (*n* = 37), medical and emergency response (*n* = 10), firefighting (*n* = 9) and law enforcement (n = 9) occupations. The most commonly reported measures included RMSSD and SDNN from time domain analysis, as well as LF, HF and the LF/HF ratio in frequency domain analysis. Five studies reported on non-linear domain measures with SD1 and SD2 being the most prominent [28–32]. The 60 articles were categorised into four distinct groups: 1) HRV comparisons in shift work and occupational tasks; 2) changes in HRV from baseline to completion of singular stressors; 3) recovery of HRV following completion of a singular stressor; 4) HRV responses across repeat stressor exposures.

### Evidence quality

The average evidence quality of included studies was high (NOS mean ± SD = 13 ± 1) with 88% (*n* = 53) considered high quality and 22% considered moderate (*n* = 7) quality (see additional file 3). 62% of studies (*n* = 37) included more than 20 participants, 37% of studies (*n* = 22) included between 10 and 20 participants and 2% included less than 10 participants (*n* = 1).

### HRV comparisons in shift work and occupational tasks

Twenty-one studies reported on measures of HRV comparing shift work and occupational tasks. Both no differences [33–36] and decreases [37] in HRV were observed in on-duty work periods compared to off-duty work periods. Twelve studies compared HRV responses to different working shift types, conditions and tasks to evaluate their similarity [2, 33, 34, 37–45]. On-duty firefighter incidents yielded lower HRV than non-incidents that were matched for similar physical activity [45]. Resting HRV was similar during comparable work shifts across a 24 h period [34, 43, 44] except in early morning shifts (3:15 am to 5:15 am compared to 5:00 am to 7:00 am) in which increases in tiredness complaints and a reduction in sleep quality occurred [43]. The increased HRV exhibited by these individuals dissipated at the onset of the new day.

Four studies observed reductions in HRV with increases in task complexity (i.e. single task vs dual task response) [40, 41, 46, 47]. Driving and piloting tasks showed an increase in HRV as the duration of the task continued [40, 48]. Decision making performance was improved with those exhibiting greater HRV both prior to and during the stressor [5, 24, 38, 46, 49]. Mentally fatigued soldier’s exhibited reduced HRV and a 16% greater error of commission (i.e. shooting when it is incorrect) while maintaining shooting accuracy compared to a controlled group [5]. Error of omission (i.e. incorrectly not shooting) was similar between groups [5]. Both decreases [40, 49] and increases [24] in HRV were associated with quicker reaction times. HRV was shown to be linked to behavioural and emotional responses in police officers and active duty infantry soldiers [39, 50]. In addition, experienced first responders demonstrated a decreased HRV compared to control subjects when completing job specific tasks [6] with increases in momentary resilience under stress coinciding with reductions in HRV [51].

### Changes in HRV from baseline to completion of singular stressors

Twenty-one studies observed HRV responses from a baseline resting period to a subsequent stress exposure with seventeen studies observing a decrease in HRV (see Table 1). From rest to during the stress exposure, time and non-linear domain HRV metrics consistently showed reductions (i.e. time domain metrics RMSSD, SDNN, PNN50 and non-linear metrics SD1, SD2) [5, 23, 28, 29, 46, 53–56, 58–62, 66]. Frequency domain variables showed less consistency with LF, LF n.u., HF n.u. and LF/HF ratio all showing mixed results of increases, decreases and no change [28, 29, 52, 54, 57, 63–65]. TP and HF appeared more consistent with both measures showing reductions [28, 53, 63, 65], or no change [52, 57, 63] from rest to stress. Of the stressors, both physical and cognitive stressors elicited a reduction in HRV from baseline to during the exposure period [53, 58, 59]. Increases in psychological hardiness correlated negatively with the change in HRV between rest and the stress exposure during a police simulation task in which participants were responding to an active shooter in a school (r = − 0.324, *p* < 0.05) [67].

**Table 1:** Changes in HRV metrics from rest to stimulus in singular stressor

			HRV Metric												
			Time Domain				Frequency Domain							Non-Linear	
Study	Participant groups	n=	RRi	RMSSD	SDNN	PNN50	TP	VLF	LF	HF	LF n.u.	HF n.u.	LF/HF	SD1	SD2
Adams et al. [52]		12	↔		↔				↔	↔			↑		
Brisinda et al. [28]		113	↓	↓	↓	↓	↓	↓ ↑%	↓ ↓%	↓ ↔%	↓	↑	↔	↓	↓
Clemente-Suárez et al. [53]		16	↔*	↓					↓	↓					
Clemente-Suárez et al. [54]		38		↓							↑	↓			
Clemente-Suárez et al. [23]		20	↓		↓										
Diaz Manzano et al. [29]	High performance	10	↓*	↓		↓					↓	↑	↓	↓	↓
	Low performance	9	↓*	↓		↓					↔	↔	↔	↓	↓
Giessing et al. [46]		19		↓											
Gnam et al. [55]		48	↓*	↓											
Hansen et al. [24]		53	↔*	↔											
Hansen et al. [56]		65		↓											
Head et al. [5]		20			↓										
Hourani et al. [57]		261							↓†	↔†					
Mulder et al. [58]		48	↓*	↓											
Saus et al. [59]		36		↓											
Souza et al. [60]		50	↓	↓											
Staller et al. [61]		25	↓*	↓											
Strahler & Ziegert [62]		50	↓*	↓											
Tornero-Aguilera & Clemente-Suárez [63]	SFV	16		↓					↑	↔					
	SNFV	16		↔					↑	↔					
	SNFNV	16		↓					↔	↔					
	Control	16		↔					↑	↓					
Tornero-Aguilera et al. [64]	Highly trained	18		↑							↑	↓			
	Low trained	22		↑							↔	↔			
	Control	9		↔							↔	↔			
Vigo et al. [65]		12	↓	↔	↓		↓†		↓†	↓†	↔	↔	↔		
Winslow et al. [66]		40			↓								↔		

Participants included (n=), *RRi* R-R interval, *RMSSD* root mean square of successive differences, *SDNN* standard deviation of R-R intervals, *PNN50* percentage of R-R intervals greater than 50 ms, *TP* total power, *VLF* very low frequency power, *LF* low frequency power, *HF* high frequency power, *LF n.u.* low frequency normative units, *HF n.u.* high frequency normative units, *LF/HF* low frequency to high frequency ratio, *SD1* standard deviation of the distance of data from the line of identity on a poincairé plot, *SD2* standard deviation of the distance of data perpendicular to the line of identity at the average RRi length on a poincairé plot,derived from heart rate (*), natural log of variable (†), percentage of variable (%), *SFV* soldier fire experience wearing night vision, *SNFV* soldiers no fire experience wearing night vision, *SNFNV* soldiers no fire experience not wearing night vision

Twenty studies looked at baseline and during stressor HRV compared to immediately post stressor measures (see Table 2). From stress to immediately post stressor, ten of eleven studies observed increases in time domain metrics of HRV [23, 24, 46, 52, 56, 58–61, 65]. Only two studies reported frequency domain metrics between stress and immediately post stressor, which did not mimic each other [52, 65]. Mixed results were observed in time and frequency domain measures when comparing baseline HRV to immediately post stressor. Of these studies, eight of fourteen reported decreases in RMSSD [58, 61, 62, 68, 70, 73–75], five of seven reported decreases in HF [69, 70, 73–75], four of seven reported increases in LF [69, 70, 74, 75], and five of nine reported increases in LF/HF ratio [30, 70–73] from baseline to immediately post the stressor. Stressors of a greater magnitude induced a greater suppression of LF and HF [76]. The change in HRV between stress exposure and the subsequent post stressor period correlated positively with psychological hardiness (r = 0.341 *p* = 0.033, r = 0.456 *p* = 0.004) and VO_2 max_ (r = 0.345, *p* = 0.027) in an active shooter police simulator task [67].

**Table 2:** HRV metrics following a singular stressor in comparison to baseline and stress time points

			HRV Metric												
Immediately post compared to stress			Time Domain				Frequency Domain							Non-Linear	
Study	Participant groups	*n*=	RRi	RMSSD	SDNN	PNN50	TP	VLF	LF	HF	LF n.u.	HF n.u.	LF/HF	SD1	SD2
Adams et al. [52]		12	↑		↑				↔	↑			↓		
Clemente-Suárez et al. [23]		20	↑		↑										
Giessing et al. [46]		19		↑											
Hansen et al. [24]		53	↑*	↑											
Hansen et al. [56]		65		↑											
Mulder et al. [58]		48	↑*	↑											
Saus et al. [59]		36		↑											
Souza et al. [60]		50	↑	↑											
Staller et al. [61]		25	↑*	↑											
Strahler & Ziegert [62]		50	↔*	↔											
Vigo et al. [65]		12	↑	↔	↑		↔†	↔† ↔%	↔† ↔%	↔† ↔%			↔		
**Immediately post compared to baseline**															
Adams et al. [52]		12	↑		↑				↔	↑			↓		
Bustamante-Sánchez & Clemente-Suárez [69]		39	↔*	↓		↔					↔	↔			
Clemente-Suárez et al. [23]		20	↓		↓										
Delgado-Moreno et al. [70]		20	↓*						↑	↓					
Delgado-Moreno et al. [71]		35		↓					↑	↓			↑		
Duarte & Morgado [72]		20	↓								↑	↓	↑		
Dussault et al. [30]	Short flight supine	7									↑	↓	↑		
	Long flight supine	26					↔				↔	↔	↔		
	Long flight standing	26	↔*	↑	↑	↔	↑				↓	↑	↓	↑	↑
Ghazali et al. [73]		48	↓			↓							↑		
Giessing et al. [46]		19		↔											
Hansen et al. [24]		53		↑											
Marins et al. [74]		13		↓					↓	↓			↑		
Mulder et al. [58]		48	↔*	↓											
Sanchez-Molina et al. [75]		19	↓*	↓					↑	↓					
Sanchez-Molina et al. [76]		19	↓*	↓					↑	↓					
Saus et al. [59]		36		↔											
Souza et al. [60]		50	↔	↔											
Staller et al. [61]		25	↓*	↓											
Strahler & Ziegert [62]		50	↓*	↓											
Vigo et al. [65]		12	↑	↔	↓		↓†	↓† ↔%	↓† ↔%	↔† ↔%			↔		

Participants included (n=), *RRi* R-R interval, *RMSSD* root mean square of successive differences, standard deviation of R-R intervals (SDNN), *PNN50* percentage of R-R intervals greater than 50 ms, *TP* total power, *VLF* very low frequency power, *LF* low frequency power, *HF*high frequency power, *LF n.u.* low frequency normative units*, HF n.u.* high frequency normative units, *LF/HF* low frequency to high frequency ratio, *SD1* standard deviation of the distance of data from the line of identity on a poincairé plot, standard deviation of the distance of data perpendicular to the line of identity at the average RRi length on a poincairé plot (SD2),derived from heart rate (*), natural log of variable (†), percentage of variable (%)

### Recovery of HRV following exposure to a singular stressor

Six studies observed changes in HRV post exposure to a single stressor (see Table 3). Time domain metrics showed a gradual return to baseline in the 2 h post the completion of stressors lasting less than 60 min [54, 62, 72]. Two studies identified sustained depressions in time domain HRV measures at 30 and 45 min post stressor before returning to baseline levels [54, 72]. Frequency domain metrics mirrored time domain metrics for the most part, with the exception of one study [72]. Dussalt et al. reported an opposite response to all other studies with a reduction in standing HRV occurring in the time course post the completion of the long flight task (~ 4.5 h) in military pilots [30]. Scheduled recovery days following a 24 h work period showed increases in HRV for rescuers [78] and ambulance personnel with many self-reported health complaints but not in those with few self-reported health complaints [77].

**Table 3:** HRV responses in subsequent recovery period after a singular stressor

Study	Timepoint of comparison		Participant groups	HRV measure											
				Time Domain				Frequency Domain						Non-Linear	
		N=		RRi	RMSSD	SDNN	PNN50	TP	LF	HF	LF n.u.	HF n.u.	LF/HF	SD1	SD2
Strahler & Ziegert [62]	20 mins post to baseline	50		↔*	↔										
	20 mins post to stress			↑*	↑										
	20 mins post to immediately post			↑*	↑										
Clemente-Suárez et al. [54]	30 mins post to baseline	38			↓						↑	↓			
	30 mins post to stress				↔						↔	↔			
Ghazali et al. [73]	45 mins post to immediately post	48		↔			↔						↔		
	75 mins post to 45 mins post			↑			↑						↔		
Dussault et al. [30]	2 & 4 h post to baseline	7	Short flight supine					↔↔			↔↔	↔↔			
			Short flight standing					↔↔					↔↑		
		26	Long flight supine		↔↔	↔↔	↔↔	↔↔			↔↔	↔↔	↔↔		
			Long flight standing	↓↓*	↔↔	↔↔	↔↔	↔↔			↔↔	↔↔	↔↑	↔↔	↔↔
	2 & 4 h post to immediately post	7	Short flight supine					↔↔			↔↔	↔↔			
			Short flight standing					↔↔					↓↔		
		26	Long flight supine	↔↔	↔↔	↔↔	↔↔	↔↔			↔↔	↔↔	↔↔		
			Long flight standing	↔↔*	↓↓	↓↓	↔↔	↓↓			↑↑	↓↓	↑↑	↓↓	↓↓
Aasa et al. [78]	work free day 1 & 2 to preceding work day	26	Few health complaints						↔↔	↔↔					
			Many health complaints						↓↓	↑↑					
Lyytikainen et al. [79]	Work free day 1, 2 & 3 to preceding work day	14	24-h recording		↑↔↑	↔↔↑							↔↓↓		
			Night time recording		↔↑↔	↔↔↔							↔↓↔		

Participants included (*n*=), *RRi* R-R interval, *RMSSD* root mean square of successive differences, *SDNN* standard deviation of R-R intervals, *PNN50* percentage of R-R intervals greater than 50 ms, *TP* total power, *VLF* very low frequency power, *LF* low frequency power, *HF* high frequency power, *LF n.u.* low frequency normative units, *HF n.u.* high frequency normative units, *LF/HF* low frequency to high frequency ratio, *SD1* standard deviation of the distance of data from the line of identity on a poincairé plot, *SD2* standard deviation of the distance of data perpendicular to the line of identity at the average RRi length on a poincairé plot, derived from heart rate (*)

### HRV responses across repeat stressor exposures

Six studies monitored HRV across repeated stress exposures with five of the studies observing increases in HRV across those exposures (see Table 4). Increases in time domain and non-linear domain metrics coincided with markers of physical adaption [79, 81], for example increased predicted VO_2 max_ [32], and the completion of military and police training courses [31, 32, 80]. Reductions in SDNN, LF and HF were observed post a six-month peacekeeping mission of Bulgarian soldiers in Kosovo, which were also lower when compared to healthy control subjects [82]. HF showed consistent increases in both supine and standing conditions with markers of physical adaptation and course completions [31, 32, 79] except in one instance [79]. LF/HF ratio, LF n.u. and HF n.u. appeared more sensitive in the supine position with increases in HF n.u. and decreases in LF/HF ratio being observed with the completion of training courses and increases in predicted VO_2 max_ [31, 32, 79–81].

**Table 4:** HRV responses in repeat stressor environments and associated findings

				HRV measure													
Supine Assessment				Time Domain				Frequency Domain							Non-Linear		Other results
Study	Notes	N=	Timepoint of comparison	RRi	RMSSD	SDNN	PNN50	TP	VLF	LF	HF	LF n.u.	HF n.u.	LF/HF	SD1	SD2	
Grant et al. [32]	20-week military training	154	12 weeks to baseline	↑	↑	↑	↑			↔	↑	↓	↑	↓	↑	↑	↑ in predicted Vo2 Max
			20 weeks to 12 weeks	↑	↑	↑	↔			↔	↑	↔	↔	↔	↑	↔	↔ in predicted Vo2 Max
George et al. [31]	9-month basic police training	60	Post to pre	↔	↑	↑	↑	↔†		↔†	↑†			↓	↑	↔	
Huovinen et al. [80]	First week of basic recruit training	24	Day 7 to day 1	↑*		↑				↑	↑	↓	↑	↔			No correlations with T:C relationship for any HRV metrics
Jouanin et al. [81]	Ranger training course	23	Post to pre	↑	↑	↑	↑	↑				↓	↑	↓			↓ plasma testosterone and body weight post course ↔ in BMI
Jouanin et al. [82]	Military mountainous training	12	Nocturnal – night 3 to night 1	↓										↓			↑ altitude sickness score on evening of day 2 compared to evening on day 1, 3 and morning on day 3 and 4
			Post effort – control to day 2 to day 3		↔ fb ↑		↓ fb ↑	↔				↑ fb ↔	↓ fb ↔	↑ fb ↔			
Nikolova et al. [83]	6-month peacekeeping mission and control group	133	Redeployment to pre-deployment	↔		↓			↔	↓	↓						
			Redeployment to control	↔		↓			↓	↓	↓						
			Pre-deployment to control	↔		↔			↓	↔	↔						
**Standing Assessment**																	
Grant et al. [32]	20-week military training	154	12 weeks to baseline	↑	↑	↑	↑			↑	↑	↔	↔	↔	↑	↑	↑ in predicted Vo2 Max
			20 weeks to 12 weeks	↑	↑	↑	↑			↔	↑	↔	↔	↔	↑	↔	↔ in predicted Vo2 Max
Huovinen et al. [80]	First week of basic recruit training	24	Day 7 to day 1	↑*		↔				↑	↔	↔	↔	↔			HR (r = −0.42), SDNN (r = 0.50), HF (r = 0.45) and HF n.u. (r = 0.47) correlated with T:C at day 7
Jouanin et al. [81]	Ranger training course	23	Post to pre	↑	↔	↑	↔	↔				↓	↑	↔			↓ plasma testosterone and body weight post course ↔ in BMI
Jouanin et al. [82]	Military mountainous training	12	Post effort – control to day 2 to day 3		↔		↔	↔				↔	↔	↔			↑ altitude sickness score on evening of day 2 compared to evening on day 1, 3 and morning on day 3 and 4

Participants included (*n*=), *HRV* heart rate variability, *RRi* R-R interval, *RMSSD* root mean square of successive differences, *SDNN* standard deviation of R-R intervals, *PNN50* percentage of R-R intervals greater than 50 ms, *TP* total power, *VLF* very low frequency power, *LF* low frequency power, *HF* high frequency power, *LF n.u.* low frequency normative units, *HF n.u.* high frequency normative units, *LF/HF* low frequency to high frequency ratio, *SD1* standard deviation of the distance of data from the line of identity on a poincairé plot, *SD2* standard deviation of the distance of data perpendicular to the line of identity at the average RRi length on a poincairé plot, *T:C* testosterone to cortisol, *BMI* body mass index, heart rate (HR), derived from heart rate (*), natural log of variable (†), followed by (fb)

## Discussion

The majority of work that has utilised HRV has been in the context of acute single-stressor exposure (see Tables 1, 2, 3); with only six studies measuring HRV in response to repeat stressor exposure (see Table 4). A reduction in HRV, via parasympathetic withdrawal and sympathetic activation, was observed in response to the onset of acute physical and cognitive occupational tasks with HRV being restored after the task’s completion. The rate of HRV restoration to baseline levels appears to be dependent on the magnitude of the stressor endured. For singular stressors, individuals of greater HRV repeatedly exhibited better decision-making performance in occupational tasks [5, 24, 38, 46, 49]. With only six studies monitoring HRV with repeated stressor exposure, more research is required to determine the chronic effects of stress and allostatic load on health and performance, and the relationships with HRV. While greater consistency in results was observed for time domain indices of HRV, the greater coefficient of variation of frequency domain metrics and their ratio values [83] requires greater participant numbers to determine their utility. As 38% (*n* = 23) of studies in this review utilised 20 participants or less, limited inferences on frequency domain metrics and their ratio values can be made. It is recommended that future research recruit larger sample sizes appropriate for the metrics being assessed.

### Changes in HRV from baseline to completion of singular stressors

In the transition from baseline to stress exposure in exercise and cognitive tasks, the expected response is a decrease in HRV as a result of parasympathetic withdrawal and sympathetic activation [84, 85]. This matches the uniform reduction in time (RMSSD, SDNN and PNN50) and non-linear domain metrics (SD1 and SD2) observed in Table 1, while frequency domain metrics showed mixed results. The frequency domain findings may be partially due to five of the seven studies that reported no change in frequency domain metrics recruited less than 20 participants. Of the time domain metrics that recorded no change in HRV, four of five studies also had less than 20 participants indicating that a lack of power may be present in determining frequency domain HRV responses. In addition, larger coefficients of variation have been reported for frequency domain measures (7–27%) and their ratios (41–82%) than time domain metrics (4–17%) which may also contribute to the lack of changes observed [83]. Three studies reported conflicting results identifying increases or no changes in RMSSD from rest to stress in soldiers and navy personnel [24, 63, 64]. This response may be explained by the presence of an anticipatory anxiety response, which has been observed elsewhere in tennis [86], where the cognitive anticipation of the task elicits a physiological stress response prior to commencement of the task, lowering baseline HRV [24, 63, 64]. The reduced HRV at baseline, due to parasympathetic downregulation, was either maintained or elevated once the task commenced and HRV increased in the period after the completion of the task due to the removal of sympathetic stimulation [24, 63, 64, 87]. The reduced parasympathetic activity at baseline is a prefrontal cortex response to regulate arousal that is followed by an increase sympathetic activity once the physical exertion task commences involving the baroreflex mechanism [88]. This response may be more relevant and prevalent in military (and law enforcement) personnel than general population individuals [63] due to their exposure to potentially fatal scenarios. This response also demonstrates HRV being sensitive to non-physical stressors. Additionally, in solely cognitive tasks, several studies have observed a decrease in HRV in transition from rest to the cognitive stressor [5, 24, 40, 49, 56]. These findings are further supported by studies demonstrating a negative association between HRV and increasing subjective job stress [89, 90] highlighting the impact of psychological stress on HRV. Therefore, individuals implementing HRV as a method for ongoing monitoring of personnel need to be aware of the factors influencing HRV when trying to interpret the data.

When transitioning from a stressor to the subsequent recovery period, increases in time domain HRV were observed in the majority of studies (see Table 2). This response is expected and consistent with responses to exercise [85] and cognitive stress [87] due to sympathetic withdrawal and parasympathetic reactivation [91]. Two studies identified no change in RMSSD, albeit one having low participant numbers (*n* = 12) [65], indicating the potential of a sustained parasympathetic withdrawal from the stress exposure that may be linked to the magnitude of the stressor [62]. In police recruits and military soldiers, increasingly stressful and complex tasks showed a greater reduction in RMSSD and SDNN [40, 41, 46], which is consistent with responses to exercise in athletic populations [92]. In addition, physical stressors of greater magnitude caused a more delayed restoration of HR and HRV metrics which influences post stressor HRV [93]. This may also explain the mixed results in comparing baseline to post-stressor measures of HRV in which RMSSD, LF, HF and LF/HF ratio observed consistent results in 55–70% of reported studies. Therefore, consideration of the stressor magnitude and any anticipatory anxiety response occurring at baseline need to be considered when comparing baseline to post stressor measurements of HRV. It is clear that HRV predominantly trends towards greater variability with the cessation of the stressor. As time domain metrics demonstrated greater consistency over frequency domain metrics in these studies, they would appear a more suitable option as markers of acute stress in the occupational setting currently. Frequency domain measures have received less exploration within these studies with some utilising low participant numbers which may mask any responses that may be present as they exhibit larger coefficients of variation [83]. Future research should look to investigate frequency domain variable responses with greater participant numbers in the acute stressor setting.

### Recovery of HRV following exposure to a singular stressor

Recordings of HRV after a single stressor exposure ranged from 15 min to 3 days post stressor. Only two studies observed HRV at the same time intervals post stressor, complicating study comparisons (see Table 3). However, it is clear that the removal of the stressful stimuli results in a gradual return of HRV metrics to baseline levels [30, 54, 62, 72, 78]. The rate of return of HRV to baseline appears to be dependent on the magnitude of the stressor individuals were exposed to. For stressor durations less than 60 min, RMSSD and PNN50 recovered at varying rates in the subsequent hour, with all returning to baseline values within 75 min [54, 62, 72]. For occupational stressors of greater duration, such as 24 h shifts, recovery of HRV to baseline levels required days rather than hours when utilising 24 h HRV recordings [77, 78]. When comparing different recording periods (five-minute resting recordings vs 24 h continuous recordings), caution should be taken as the different methods may produce different results. The use of 24 h continuous recordings fail to account for factors such as increased physical activity which has been shown to reduce HRV [2, 33]. As 24 h continuous recordings don’t lend themselves to identical recording conditions, these aspects can change the apparent HRV metrics derived, particularly if there are different amounts of movement or exercise on different days [33, 51]. One studies (*n* = 12) that observed HRV across a 24 h period including night shift observed an increase in the LF/HF ratio along with no changes in LF or HF when transitioning from pre to during the shift, indicating a change towards greater sympathetic stimulation. Typically, frequency ratio values exhibit greater variation the frequency domain variables [83]. As this study utilised twelve consecutive, five-minute segments to analysis both baseline and during shift HRV, greater variations in LF and HF occurred throughout the 2 h while proportions of sympathetic and parasympathetic activation remained similar [52]. Therefore, the use of ratio metrics such as LF n.u., HF n.u. and LF/HF ratio may be more suited as an observation and comparison of the amount of stress individuals experience across days or shifts using longer continuous recordings. What should be taken from these studies is that HRV appears sensitive to the magnitude of stressors experienced in first responders and tactical operators that can indicate the residual stress on individuals after occupational tasks, and potential readiness for subsequent tasks or shifts.

### HRV relationships with occupational performance and fatigue

Of particular interest to tactical operators and first responders is the relationship between HRV and occupational performance. Soldiers and navy personnel with greater HRV, both at rest and under stress, exhibited better performance in decision making and cognitive tasks, for example threat discrimination shooting tasks [5, 24, 38, 40, 49]. An essential requirement of these occupations is decision making in highly stressful scenarios. In particular Head et al. identified an increased error of commission in shooting responses (i.e. shooting when it is incorrect) of mentally fatigued individuals who exhibited lower HRV; however, no changes were seen in shot accuracy or error of omission, (i.e. incorrectly not shooting) [5]. This poses as an issue as the mentally fatigued individuals with lower HRV maintained their lethality, as demonstrated by their accuracy, when shooting at incorrect targets. The authors identify the high error of commission in both conditions (48% for fatigue, 32% for control) as a result of the high quantity of ‘Go’ responses providing a target rich environment in which participants exhibit a failure of inhibitory control [5]. In sport, scenarios requiring decisions of greater consequence, that are indicative of higher anxiety, have been shown to impair decision making performance [94] which may be particularly relevant to this target rich shooting task. It may indicate that HRV could be used as a potential identifier of poorer decision-making capacity in this context. Observations into the performance of different cognitive tasks have identified different HRV responses occur irrespective of physical exertion, highlighting the complex and dynamic interplay of parasympathetic and sympathetic activity that is required [88, 95]. In addition, emotional and behavioural responses to situations can alter the level of stress depicted by HRV, highlighting inter-individual differences in stress responses [39, 50, 67]. An individual’s psychological hardiness has also correlated with changes in HRV indicating some individuals may be better equipped to deal and recover from stressful incidents [67]. While HRV may provide great utility in managing first responders and tactical personnel, each of these studies examined decision making or cognitive performance at a single timepoint that may be affected by these inter-individual differences. Future research would benefit from investigating whether within subject changes in HRV could predict changes in these performance outcomes. Nevertheless, it indicates a relationship between the two variables that may render HRV as a useful monitoring tool for cognitive performance capacity in these occupations if supported by future studies adopting within subject designs.

Of the studies that met the inclusion criteria, fifty-eight of the sixty studies involved tasks that contribute to fatigue as a result from stressors and potentially inadequate recovery. In contrast, two papers reported the opposing response whereby fatigue results from passive driving and piloting tasks that exhibit a lack of stimulation [30, 48]. Military truck drivers undergoing a driving simulation exhibited increases in HRV and fatigue, which showed a moderate correlation (r = 0.32, *p* = 0.05), as the simulation continued until the test was completed [48]. Increases in sleepiness scores were also observed leading to the test completion that was a result of participants falling asleep at the wheel or being unable to continue, with the average driving duration of the test being 92 ± 14 min [48]. Additionally, increases in HRV were observed post a long air support flight (~ 4.5 h) with standing HRV returning back to baseline levels by 2 h after the flight completion, indicating a recovery from the parasympathetic shift caused by the task [30]. Tasks of long duration may lead to increased parasympathetic activation acting as a calming response as increases in time on task are associated with increased subjective fatigue and HRV [96]. These effects may be particularly prevalent in nocturnal shift work in which greater HRV was accompanied by increased ratings of sleepiness that could compromise occupation performance [22, 43]. This may be linked to circadian rhythm in which a shift towards greater parasympathetic predominance occurs at night [21] and/or related to a change in sympathetic-parasympathetic balance related to increases in sleepiness [22]. While this review is focusing on HRV responses to sympathetic stressors it is important to consider the opposing response in which HRV may provide a useful monitoring tool for passive fatigue that results from certain driving and piloting tasks in these occupations. Further research should investigate the utility of HRV to mitigate the risk of fatigue related accidents.

In summary, using HRV as a measure to monitor acute allostatic load following exposure to a single acute stressor appears suitable with a few key considerations. It has shown to be sensitive to both physical [53] and cognitive stressors [40], and provides an indication of the magnitude of stress exposure or internal stress on an individual. Therefore, it is important to take into consideration the recording environment/s and scenario/s when utilising HRV to monitor personnel, as it can affect HRV and the interpretations when comparing analysis periods. The rate of recovery of HRV metrics post stimulus appears to provide an indication of the changes in strain over time, which can be useful for monitoring recovery in first responders and tactical personnel. The apparent sensitivity of HRV to both sympathetic stress and driving/piloting fatigue reinforces the potential utility of HRV in these occupations. Currently, it appears that more consistent responses are seen in time (RMSSD, SDNN and PNN50) and non-linear domain metrics (SD1 and SD2) of HRV compared with frequency domain measures in response to an acute stressor. However, further investigations into frequency domain measures are warranted as low participant numbers were utilised in papers that observed no changed in these metrics. Furthermore, HF power showed the greatest consistency in HRV changes between baseline and immediately post stressor, which aligned with RMSSD.

### HRV responses across repeat stressor exposure

From the six studies reporting on HRV responses to repeat stressor exposure there is insufficient data available to determine whether HRV is an appropriate measure of physiological status in first responders and tactical operators. The results from the included papers suggest that: i) increases in HRV generally, but not always, coincide with markers of physical adaption and/or the removal of stressors; ii) pre-post HRV assessment across a program is not suitable for evaluating the utility of HRV as a monitoring tool of allostatic load and physiological status. To date, only one study has assessed daily resting HRV with changes in other markers of stress to give a depiction of what these daily changes mean [79]. This can make potential explanations difficult and highlights that further research into the suitability of HRV as a repeated resting measure of allostatic load and physiological status is still required if it is to be used as a monitoring tool.

Of the six studies that reported on repeat stress events, four of these studies reported increases in time domain HRV indices RMSSD, SDNN, RRi and PNN50 [31, 32, 79, 80]**.** These studies identify HRV increases at the completion of a training period or course [31, 32, 79, 80] and some are identified with other markers of physical adaption (i.e. increases in cardiorespiratory fitness and the testosterone to cortisol relationship) [32, 79]. Increases in HRV have been observed with increases in cardiorespiratory fitness in these populations indicating an adapted state [35, 36, 67]. Increases in HRV observed at the completion of training courses may be due to the reduced allostatic load as a result of the course completion, similar to the responses observed in the acute setting.

In contrast, decreases in HRV were observed in Bulgarian soldiers returning from a six-month peacekeeping mission [82]. The cause of this response in unknown but may involve some aspects of stress with returning home or potential reductions in cardiorespiratory fitness [97]. The lack of research repeatedly measuring HRV across these programs leaves these hypotheses to be further tested. Furthermore, increases in HRV have also been observed at the completion of a ranger training course in military cadets along with decreases in plasma testosterone (~ 28%) and body weight (~ 1.1 kg) [80]. Decreases in testosterone are normally associated with increased fatigue and poor recovery which is the opposite of what is expected of increased HRV [10, 33]. Looking at a more granular data set investigating soldier HRV responses to exercise and altitude acclimatisation, a reduction and subsequent super compensatory increase in some HRV indices were observed over a three-day period [81]. Due to a low number of participants and the inter-individual variation in HRV indices, only some variables show significant changes, however similar responses have been observed in sport settings [98] and with increasing altitude exposure [99]. These findings suggest that there may be a more complex HRV response following repeat stressor exposures than what is observed in the acute stressor setting, and further research is required to better understand this response.

## Conclusions

In summary, the use of HRV to monitor acute stress and recovery responses to tasks of first responders and tactical operators appears sensitive and suitable. Research into the suitability of HRV as a monitoring tool of chronic allostatic load in first responders and emergency service personnel is currently not sufficient for inferences to be made. Future research should look to repeatedly assess HRV with sufficient power in these environments along with other markers of physical, psychological and cognitive stress to determine whether HRV can be used as a viable marker of allostatic load in these contexts. Understanding how HRV relates to job specific performance in a within subject design is also required. Current evidence shows that it is important that practitioners looking to use HRV need to be conscious of the manner and context in which it is recorded when interpreting results.

## Supplementary Information

**Additional file 1.**

**Additional file 2.**

**Additional file 3.**

## Data Availability

All data included in this review is available from the referenced articles.

## Abbreviations

HF: High frequency power
HRV: Heart rate variability
LF: Low frequency power
LF/HF ratio: Low frequency to high frequency ratio
n.u.: Normative unit
PNN50: Percentage of R-R intervals greater than 50 ms
PRISMA: Preferred reporting items for systematic reviews and meta-analyses
RMSSD: Root mean square of successive differences of R-R intervals
RRi: R-R interval
SD: Standard deviation
SDNN: Standard deviation of R-R intervals
SD1: Standard deviation of data perpendicular to y = x line on poincairé plot
SD2: Standard deviation of data on y = x line on poincairé plot
TP: Total power
VLF: Very low frequency power
