# Supplementary material for: Monitoring stress and allostatic load in first responders and tactical operators using heart rate variability: a systematic review
Source: BMC Public Health. 2021 Sep 18;21:1701. doi: 10.1186/s12889-021-11595-x (PMC8449887; doi:10.1186/s12889-021-11595-x)
Supplement: Supplementary file 1 — Additional file 1. [file 12889_2021_11595_MOESM1_ESM.docx]

Supplementary File – Final Search Queries

Scopus

## ( ( ( TITLE-ABS ( ambulance AND personnel ) OR TITLE-ABS ( armed AND forces ) OR TITLE-ABS ( army ) OR TITLE-ABS ( defence AND force* ) OR TITLE-ABS ( emt ) OR TITLE-ABS ( firefighters ) OR TITLE-ABS ( fire-fighters ) OR TITLE-ABS ( fire AND fighters ) OR TITLE-ABS ( first AND respond* ) OR TITLE-ABS ( police* ) OR TITLE-ABS ( law AND enforcement ) OR TITLE-ABS ( military ) OR TITLE-ABS ( navy ) OR TITLE-ABS ( paramedic* ) OR TITLE-ABS ( soldiers ) OR TITLE-ABS ( troops ) OR TITLE-ABS ( Air Force ) OR TITLE-ABS ( pilot ) ) AND DOCTYPE ( ar ) AND PUBYEAR > 1984 ) AND ( ( TITLE-ABS ( fatigue* ) OR TITLE-ABS ( load* ) OR TITLE-ABS ( stress ) OR TITLE-ABS ( overtrain* ) OR TITLE-ABS ( overreach* ) OR TITLE-ABS ( recovery ) ) AND DOCTYPE ( ar ) AND PUBYEAR > 1984 ) AND ( ( TITLE-ABS ( ans ) OR TITLE-ABS ( autonomic ) OR TITLE-ABS ( pns ) OR TITLE-ABS ( parasympathetic ) OR TITLE-ABS ( sns ) OR TITLE-ABS ( sympathetic ) OR TITLE-ABS ( vagal ) OR TITLE-ABS ( heart AND rate AND variability ) OR TITLE-ABS ( hrv ) ) AND DOCTYPE ( ar ) AND PUBYEAR > 1984 ) AND  ( LIMIT-TO ( LANGUAGE ,  "English" ) )

Academic Search Complete, Medline Complete, APA PsycInfo, SPORTDiscus

( TI ( heart rate variability OR hrv OR autonomic OR ans OR pns OR parasympathetic OR sns OR sympathetic OR vagal ) OR AB ( heart rate variability OR hrv OR autonomic OR ans OR pns OR parasympathetic OR sns OR sympathetic OR vagal ) ) AND ( TI ( fatigue OR load* OR stress OR overtrain* OR overreach* OR recovery ) OR AB ( fatigue OR load* OR stress OR overtrain* OR overreach* OR recovery ) ) AND ( TI ( ambulance personnel OR armed forces OR army OR soldiers OR defence force* OR emt OR firefighters OR fire fighters OR navy OR paramedic* OR first respond* OR police* OR law enforcement OR military OR troops OR Air Force OR pilot ) OR AB ( ambulance personnel OR armed forces OR army OR soldiers OR defence force* OR emt OR firefighters OR fire fighters OR navy OR paramedic* OR first respond* OR police* OR law enforcement OR military OR troops OR navy OR Air Force OR pilot) )

Limiters: Peer Reviewed, English Language, 1/1/1985 – 3/8/2020

Pub Med

(("ambulance personnel"[Title/Abstract] OR "armed forces"[Title/Abstract] OR "army"[Title/Abstract] OR "soldiers"[Title/Abstract] OR "defence force*"[Title/Abstract] OR "emt"[Title/Abstract] OR "firefighters"[Title/Abstract] OR "fire fighters"[Title/Abstract] OR "navy"[Title/Abstract] OR "paramedic*"[Title/Abstract] OR "first respond*"[Title/Abstract] OR "police*"[Title/Abstract] OR "law enforcement"[Title/Abstract] OR "military"[Title/Abstract] OR "troops"[Title/Abstract] OR "air force"[Title/Abstract] OR "pilot"[Title/Abstract]) AND ("journal article"[Publication Type] AND "loattrfull text"[Filter] AND "english"[Language] AND 1985/01/01:2020/8/3[Date - Publication]) AND ("journal article"[Publication Type] AND "loattrfull text"[Filter] AND "english"[Language]) AND ("fatigue"[Title/Abstract] OR "load*"[Title/Abstract] OR "stress"[Title/Abstract] OR "overtrain*"[Title/Abstract] OR "overreach*"[Title/Abstract] OR "recovery"[Title/Abstract]) AND ("journal article"[Publication Type] AND "loattrfull text"[Filter] AND "english"[Language] AND 1985/01/01:2020/8/3[Date - Publication]) AND (("heart rate variability"[Title/Abstract] OR "autonomic"[Title/Abstract] OR "ans"[Title/Abstract] OR "pns"[Title/Abstract] OR "parasympathetic"[Title/Abstract] OR "hrv"[Title/Abstract] OR "sns"[Title/Abstract] OR "sympathetic"[Title/Abstract] OR "vagal"[Title/Abstract]) AND ("journal article"[Publication Type] AND "loattrfull text"[Filter] AND "english"[Language] AND 1985/01/01:2020/8/3[Date - Publication]))) AND ((journalarticle[Filter]) AND (fft[Filter]) AND (english[Filter]))
