# Supplementary material for: Monitoring stress and allostatic load in first responders and tactical operators using heart rate variability: a systematic review
Source: BMC Public Health. 2021 Sep 18;21:1701. doi: 10.1186/s12889-021-11595-x (PMC8449887; doi:10.1186/s12889-021-11595-x)
Supplement: Supplementary file 2 — Additional file 2. [file 12889_2021_11595_MOESM2_ESM.docx]

Criteria for Quality Assessment

| Criteria | | Definition | Score | | |
| --- | --- | --- | --- | --- | --- |
|  |  |  | 0 | 1 | 2 |
| 1 | Number of participants | Number of participants contributing to study findings | <10 | 10-20 | >20 |
| 2 | Population defined | Age and gender of participants | No | Partially | Yes |
| 3 | Occupation status | Do the participants have a role as a first responder or tactical operator (detailed in search terms) | Not detailed | No, but completing the tasks of these occupations in the study | Yes |
| 4 | Measurement tool used for HRV recording | Is the apparatus used for HRV recording valid | No | Validity and Reliability not established | Valid and Reliable |
| 5 | Time points of HRV recording | Instances in which HRV is recorded | 0 | 1-2 | >2 |
| 6 | Detail of stress/stimulus defined | Stimulus applied to participants is clearly described | No | Partially | Yes |
| 7 | Repeatable measurement protocol | Methods are reproduced | No | Partially | Yes |
