# Supplementary material for: Monitoring stress and allostatic load in first responders and tactical operators using heart rate variability: a systematic review
Source: BMC Public Health. 2021 Sep 18;21:1701. doi: 10.1186/s12889-021-11595-x (PMC8449887; doi:10.1186/s12889-021-11595-x)
Supplement: Supplementary file 3 — Additional file 3. [file 12889_2021_11595_MOESM3_ESM.docx]

Evidence of Quality Appraisal

| **Author** | **Year** | **Number of participants** | **Population defined** | **Occupation Status** | **Measurement tool used for HRV recording** | **Time points of HRV recording** | **Detail of stress/stimulus defined** | **Repeatable measurement protocol** | **Total** | **Quality Category** | | |
| --- | --- | --- | --- | --- | --- | --- | --- | --- | --- | --- | --- | --- |
|  |  |  |  |  |  |  |  |  |  | **High (>11)** | **Medium (8-11)** | **Low (<8)** |
| Aasa et al. (78) | 2006 | 2 | 2 | 2 | 2 | 2 | 2 | 2 | 14 | 1 | - | - |
| Adams et al. (62) | 1998 | 1 | 2 | 2 | 2 | 2 | 2 | 2 | 13 | 1 | - | - |
| Brisinda et al. (28) | 2015 | 2 | 2 | 2 | 2 | 1 | 2 | 1 | 12 | 1 | - | - |
| Bustamante-Sánchez &  Clemente-Suárez (68) | 2020 | 2 | 2 | 2 | 2 | 1 | 2 | 1 | 12 | 1 | - | - |
| Clemente-Suarez et al. (23) | 2018 | 1 | 2 | 2 | 2 | 1 | 2 | 2 | 12 | 1 | - | - |
| Clemente-Suarez et al. (52) | 2017 | 2 | 2 | 2 | 2 | 2 | 2 | 2 | 14 | 1 | - | - |
| Clemente-Suarez et al. (53) | 2017 | 1 | 2 | 2 | 2 | 1 | 2 | 2 | 12 | 1 | - | - |
| Clifford et al. (47) | 2020 | 2 | 1 | 2 | 2 | 2 | 2 | 2 | 13 | 1 | - | - |
| Delgado Moreno et al. (69) | 2019 | 2 | 2 | 2 | 2 | 1 | 2 | 2 | 13 | 1 | - | - |
| Delgado-Moreno et al. (73) | 2017 | 1 | 2 | 2 | 2 | 1 | 2 | 2 | 12 | 1 | - | - |
| Diaz Manzano et al. (29) | 2018 | 1 | 2 | 2 | 2 | 1 | 2 | 2 | 12 | 1 | - | - |
| Duarte & Morgado (74) | 2015 | 1 | 2 | 2 | 2 | 1 | 1 | 1 | 10 | - | 1 | - |
| Dussault et al. (30) | 2009 | 2 | 2 | 2 | 2 | 2 | 2 | 2 | 14 | 1 | - | - |
| Fogt et al. (35) | 2009 | 2 | 2 | 2 | 2 | 1 | 2 | 2 | 13 | 1 | - | - |
| Gamble et al. (38) | 2018 | 2 | 2 | 2 | 2 | 1 | 2 | 2 | 13 | 1 | - | - |
| George et al. (31) | 2018 | 2 | 2 | 2 | 2 | 1 | 1 | 2 | 12 | 1 | - | - |
| Ghazali et al. (75) | 2018 | 2 | 2 | 2 | 0 | 2 | 2 | 2 | 12 | 1 | - | - |
| Giessing et al. (46) | 2019 | 1 | 2 | 2 | 2 | 2 | 2 | 2 | 13 | 1 | - | - |
| Gnam et al. (54) | 2018 | 2 | 2 | 2 | 2 | 1 | 2 | 2 | 13 | 1 | - | - |
| Grant et al. (32) | 2016 | 2 | 2 | 2 | 2 | 2 | 2 | 2 | 14 | 1 | - | - |
| Haller et al. (39) | 2014 | 2 | 2 | 2 | 2 | 2 | 2 | 2 | 14 | 1 | - | - |
| Hansen & Johnsen (55) | 2013 | 2 | 2 | 2 | 2 | 2 | 2 | 2 | 14 | 1 | - | - |
| Hansen et al. (24) | 2003 | 2 | 2 | 2 | 2 | 2 | 2 | 2 | 14 | 1 | - | - |
| Hansen et al. (49) | 2009 | 2 | 2 | 2 | 2 | 1 | 2 | 2 | 13 | 1 | - | - |
| Head et al. (5) | 2017 | 1 | 1 | 2 | 2 | 1 | 2 | 2 | 11 | - | 1 | - |
| Head et al. (40) | 2019 | 1 | 2 | 2 | 2 | 2 | 2 | 2 | 13 | 1 | - | - |
| Hourani et al. (63) | 2020 | 2 | 2 | 2 | 2 | 2 | 2 | 2 | 14 | 1 | - | - |
| Huovinen et al. (79) | 2009 | 2 | 2 | 2 | 2 | 2 | 1 | 2 | 13 | 1 | - | - |
| Jouanin et al. (80) | 2009 | 1 | 2 | 2 | 2 | 2 | 2 | 2 | 13 | 1 | - | - |
| Jouanin et al. (81) | 2004 | 2 | 2 | 2 | 2 | 1 | 1 | 2 | 12 | 1 | - | - |
| Kaikkonen et al. (2) | 2017 | 2 | 2 | 2 | 2 | 1 | 2 | 2 | 13 | 1 | - | - |
| Lyytikainen et al. (77) | 2017 | 1 | 2 | 2 | 2 | 2 | 2 | 2 | 13 | 1 | - | - |
| Marins et al. (70) | 2020 | 1 | 2 | 2 | 2 | 1 | 2 | 2 | 12 | 1 | - | - |
| Matthews et al. (41) | 2015 | 2 | 2 | 1 | 2 | 2 | 2 | 2 | 13 | 1 | - | - |
| Meina et al. (45) | 2020 | 2 | 2 | 2 | 2 | 1 | 2 | 2 | 13 | 1 | - | - |
| Mulder et al. (59) | 2020 | 2 | 2 | 2 | 2 | 2 | 2 | 2 | 14 | 1 | - | - |
| Nikolova et al. (82) | 2007 | 2 | 2 | 2 | 2 | 1 | 2 | 1 | 12 | 1 | - | - |
| Oldenburg et al. (6) | 2014 | 2 | 2 | 2 | 2 | 1 | 2 | 2 | 13 | 1 | - | - |
| Oron-Gilad & Ronen (47) | 2007 | 1 | 2 | 2 | 2 | 2 | 2 | 2 | 13 | 1 | - | - |
| Petrowski et al. (36) | 2019 | 1 | 2 | 2 | 2 | 2 | 2 | 2 | 13 | 1 | - | - |
| Pierce et al. (42) | 2018 | 1 | 1 | 2 | 2 | 1 | 2 | 1 | 10 | - | 1 | - |
| Porto et al. (36) | 2019 | 2 | 2 | 2 | 2 | 2 | 2 | 2 | 14 | 1 | - | - |
| Prell et al. (76) | 2020 | 1 | 2 | 2 | 2 | 2 | 2 | 2 | 13 | 1 | - | - |
| Roy et al. (50) | 2019 | 2 | 2 | 2 | 2 | 1 | 2 | 2 | 13 | 1 | - | - |
| Sanchez-Molina et al. (71) | 2017 | 1 | 2 | 2 | 2 | 1 | 2 | 2 | 12 | 1 | - | - |
| Sanchez-Molina et al. (72) | 2018 | 1 | 2 | 2 | 2 | 1 | 2 | 2 | 12 | 1 | - | - |
| Sandvik et al. (67) | 2019 | 2 | 2 | 2 | 2 | 2 | 2 | 2 | 14 | 1 | - | - |
| Saus et al. (56) | 2012 | 2 | 2 | 2 | 2 | 2 | 2 | 2 | 14 | 1 | - | - |
| Schwerdtfeger & Dick (51) | 2018 | 2 | 2 | 2 | 2 | 1 | 2 | 2 | 13 | 1 | - | - |
| Souza et al. (57) | 2013 | 2 | 2 | 2 | 2 | 2 | 2 | 2 | 14 | 1 | - | - |
| Staller et al. (60) | 2019 | 2 | 2 | 2 | 0 | 2 | 2 | 1 | 11 | - | 1 | - |
| Strahler & Ziegert (58) | 2015 | 2 | 2 | 2 | 2 | 2 | 2 | 2 | 14 | 1 | - | - |
| Suzuki et al. (33) | 2016 | 0 | 2 | 2 | 2 | 1 | 2 | 2 | 11 | - | 1 | - |
| Takeyama et al. (43) | 2005 | 1 | 1 | 2 | 2 | 2 | 2 | 1 | 11 | - | 1 | - |
| Takeyama et al. (44) | 2009 | 1 | 1 | 2 | 2 | 2 | 2 | 2 | 12 | 1 | - | - |
| Tornero-Aguilera &  Clemente-Suárez (64) | 2018 | 1 | 2 | 2 | 2 | 2 | 2 | 1 | 12 | 1 | - | - |
| Tornero-Aguilera et al. (65) | 2018 | 2 | 1 | 2 | 2 | 1 | 2 | 1 | 11 | - | 1 | - |
| Vigo et al. (66) | 2010 | 1 | 2 | 2 | 2 | 2 | 2 | 2 | 13 | 1 | - | - |
| Winslow et al. (61) | 2015 | 2 | 2 | 1 | 2 | 2 | 2 | 2 | 13 | 1 | - | - |
| Wong et al. (34) | 2012 | 2 | 2 | 2 | 2 | 1 | 2 | 2 | 13 | 1 | - | - |
| **Total** |  |  |  |  |  |  |  |  |  | **53** | **7** | **0** |
| **Mean (SD)** |  |  |  |  |  |  |  |  | **13(1)** |  |  |  |
